# A Social Disruptiveness-Based Approach to AI Governance: Complementing the Risk-Based Approach of the AI Act

**DOI:** 10.1007/s11948-025-00545-0

**Published:** 2025-08-27

**Authors:** Samuela Marchiori, Jeroen K. G. Hopster, Anna Puzio, M. Birna van Riemsdijk, Steven R. Kraaijeveld, Björn Lundgren, Juri Viehoff, Lily E. Frank

**Affiliations:** 1 Department of Values, Technology and Innovation, Delft University of Technology, Delft, The Netherlands; 2 Department of Philosophy and Religious Studies, Utrecht University, Utrecht, The Netherlands; 3 Faculty of Behavioural, Management and Social Sciences, Philosophy, University of Twente, Enschede, The Netherlands; 4 Human Media Interaction Department, University of Twente, Enschede, The Netherlands; 5 Amsterdam UMC, Department of Ethics, Law & Medical Humanities, Amsterdam, The Netherlands; 6 Centre for Philosophy and AI Research, Friedrich-Alexander-Universität Erlangen-Nürnberg, Erlangen, Germany; 7 Institute for Futures Studies, Stockholm, Sweden; 8 Department of Philosophy and Religious Studies, Utrecht University, Utrecht, The Netherlands; 9 School of Industrial Engineering and Innovation Science, Department of Philosophy and Ethics, Eindhoven University of Technology, Eindhoven, The Netherlands

**Keywords:** Artificial Intelligence (AI), AI act, Governance, Social disruption, Socially disruptive technologies, Responsible Research and Innovation (RRI)

## Abstract

The AI Act advances a risk-based approach to the legal regulation of AI systems in the European Union. While we support this development, we argue that adequate AI governance requires paying attention to the broader implications of AI systems on the socio-technical landscape in which they are designed, developed, and used. In addition to risk-based impact assessments, this involves coming to terms with the socially disruptive implications of AI, which should be governed and guided in a dynamic ecosystem of regulation, law, ethics, and evolving human practice. In this paper, we outline a ‘social disruptiveness-based’ approach to AI governance aimed at addressing disruptions by AI that are not easily captured by legal regulation, but that are nonetheless of great societal and ethical concern. We argue that integrating the AI Act risk-based approach with a social disruptiveness-based approach can offer a more nuanced understanding of the dimensions of impact of AI systems on society at large, thus enhancing the governance of AI and other socially disruptive technologies.

## Introduction

Artificial intelligence’s (AI) wide-ranging impacts call for a critical examination of the ramifications of the design, development, and use of AI systems, and underscores the pressing need for the development of a robust framework for the ethical governance of AI (European Commission, 2020; McLennan et al., 2020; Winfield & Jirotka, 2018). The AI Act provides a regulatory framework to address some of these concerns in the form of a proportionate risk-based approach to the regulation of AI in the European Union (EU). AI systems and practices deemed highly or unacceptably risky are subject to stricter requirements compared to AI systems and practices that are considered minimally risky.

While legal guardrails can provide a solid backbone for good AI governance, neither the Act nor legal regulation at large can alone exhaust measures for the governance of AI (Bullock et al., 2024). This proposition gets support from the scholarly literature on Responsible Research and Innovation (RRI) (von Schomberg, 2013; Dignum, 2019), and has long been appreciated in ELSA (Ethical, Legal, and Social Aspects; e.g., Forsberg, 2015) and GELSI (Governance, Ethical, Legal and Social Implications; e.g., Ghioni et al., 2023) approaches to technology assessment and governance, on which the wider EU AI policy framework (the so-called ‘ecosystem of excellence’) is based. These approaches underscore that it is unrealistic to place all the burdens of AI governance on legal regulation, and that over-reliance on legal measures can even be harmful. Rather, legal regulation should be understood as one of several facets of good AI governance, and should be complemented with non-legal measures to build a resilient ecosystem of regulation, ethics, and human practice to facilitate and guide the responsible design, development, deployment, and use of AI (Hopster & Maas, 2023; Novelli et al., 2024).

In this paper, we build on the insights of RRI, ELSA, and GELSI approaches to argue that the AI Act should be complemented with additional governance measures aimed at addressing disruptions by AI that are not easily captured by legal regulation, but that are nonetheless of great societal and ethical concern. In particular, we argue that the risk-based approach promoted by the AI Act needs a complementary ‘social disruptiveness-based approach’. This approach, which has recently been developed in the field of philosophy of technology, can be used to triage technological developments with a marked potential to yield transformative and disorienting social and moral changes (Hopster, 2021b; Gruetzemacher & Whittlestone, 2022), such as the large-scale implementation of AI systems (see Sect. 3.5). We argue that this perspective should be considered as an important counterpart to the AI Act’s risk-based approach.

The paper is structured as follows. Section two delves into the AI Act’s proportionate risk-based approach, examining how AI systems and practices are currently understood and regulated through the lenses of the notion of risk within the legislative framework of the European Union. It also highlights some concerns with such a risk-based approach as it regards the soft impacts of AI systems, which such an approach fails to capture. Section three builds on these insights and introduces the social disruptiveness-based approach, outlining its key concepts and the importance of addressing AI’s transformative implications on society beyond mere risk assessments. Specifically, this section explores the features and benefits of a social disruptiveness-based approach, by illustrating that social disruptiveness can manifest along several dimensions of impact and in different degrees. It furthermore demonstrates how such a framework can be operationalised and generalised beyond AI systems to include socially disruptive technologies at large. Lastly, section four contains our concluding remarks. Here, we synthesise our argument, emphasising the importance of complementing the AI Act with non-legal measures to foster a comprehensive ecosystem of AI governance.

### Generalisability. Socially Disruptive Technologies

The social disruptiveness-based approach is applicable to AI and to Socially Disruptive Technologies (SDTs) more broadly (Carlsen et al., 2010; Hopster, 2021b). By SDTs, we mean emerging technologies that contribute to substantial challenges and require new ways of thinking about norms (normative reorientation) in a given societal domain or in society at large [reference removed for blind review]. Examples of SDTs range from brain chips to quantum computers. They are not connected by their underlying techniques, but by their potential to transform human society, nature, and foundational aspects of the human condition.

The transformative potential of AI has become evident in recent years. For instance, the rise of generative AI systems has provoked debate about the nature and value of fundamental human capacities such as ‘creativity’ and artistic creation (Astola et al., 2022; Rafner et al., 2023; Kraaijeveld, 2024), ‘intelligence’ (Cave, 2020; Gebru & Torres, 2024), and ‘understanding’ (Bender & Koller, 2020). It also challenges important social and legal concepts, values and institutions, such as ‘authorship’ and ‘ownership’ (Smits & Borghuis, 2022; Jiang et al., 2023). Each of these concepts express values or are associated with an entrenched set of (legal and social) norms, such as intellectual property rights, the unreflective application of which is contested or cast in doubt because of new affordances and incentives generated by emerging AI applications.

In recent years, the academic scholarship on SDTs has experienced a sharp increase (Hopster, 2021a, b; van de Poel, 2022a; van de Poel et al., 2023; Giovanola, 2023; Hermann, 2023), not in small part due to explicit attention being placed on SDTs by research programs such as Ethics of Socially Disruptive Technologies (ESDiT), whose aim is to “develop a comprehensive philosophical understanding of the socially disruptive technologies of the 21st century, and develop new moral frameworks to guide [such technologies]” (ESDiT^1^). A key part of such efforts includes a conceptual engineering approach to the philosophy of SDTs, which seeks to (advance methods and frameworks to) evaluate, design, revise, replace, or implement concepts in response to technology-induced conceptual disruptions (Löhr, 2023a, b; Hopster, 2024; Marchiori & Scharp, 2024). This move to conceptual engineering is a natural response to the ‘hard problems’ provoked by SDTs (Hopster & Maas, 2023), which cast doubt on the adequacy of pre-existent ethical principles, norms, values, codes, regulations, and law, calling for foundational conceptual reflection in response.

We anticipate that a social disruptiveness-based approach to the ethical governance of technology will prove beneficial to a broader set of technologies beyond artificial intelligence systems. In principle, it can be applied to any technology that plausibly satisfies criteria for social disruptiveness (e.g. quantum computing, solar geo-engineering, implantable brain-computer interfaces, etc. – see Sect. “Social Disruptiveness-Based Approach” for discussion of these criteria).

## The AI Act’s Proportionate Risk-Based Approach

Before introducing the social disruptiveness-based approach, we must first examine how risk is currently treated in the AI Act. The AI Act adopts a proportionate risk-based approach to the regulation of AI in the EU, inspired by previous work commissioned by the EU (High Level Expert Group on AI, 2019; European Commission, 2020, 2021; European Commission Staff Working Document, 2021) (see Table 1).

**Table 1:** Overview of risk-based approach in AI act and preparatory work

Ethics Guidelines for Trustworthy AI (High Level Expert Group on AI, 2019)	“While offering great opportunities, AI systems also give rise to certain risks that must be handled appropriately and proportionately” (*p*. 4, emphasis added)
White Paper on AI (European Commission, 2020)	“[W]hen designing the future regulatory framework for AI, it will be necessary to decide on the types of mandatory legal requirements to be imposed on the relevant actors. […] [T]hose requirements would apply to *high-risk* AI applications only, thus ensuring that any regulatory intervention is focused and *proportionate*” (emphasis added)
Impact Assessment (European Commission Staff Working Document, 2021)	“Option 3: Horizontal EU legislative instrument following a *proportionate risk-based approach*; Option 3+: Horizontal EU legislative instrument following a *proportionate risk-based approac*h + codes of conduct for non-high-risk AI systems” (emphasis added)
Commission’s Proposal (European Commission, 2021)	“[T]he preferred option is option 3+, namely a regulatory framework for high-risk AI systems only, with the possibility for all providers of non-high-risk AI systems to follow a code of conduct” (p. 4)
Artificial Intelligence Act	“In order to introduce a *proportionate* and effective set of binding rules for AI systems, *a clearly defined risk-based approach should be followed*. That approach should tailor the type and content of such rules to the intensity and scope of the risks that AI systems can generate. It is therefore necessary to prohibit certain unacceptable AI practices, to lay down requirements for high-risk AI systems and obligations for the relevant operators, and to lay down transparency obligations for certain AI systems” (Recital 26, emphasis added)

In the Act, ‘risk’ is defined in Article 3(2) as “the combination of the probability of an occurrence of harm and the severity of that harm”, where ‘harm’ “might be material or immaterial, including physical, psychological, societal or economic harm” (Recital 5). Four risk categories can be identified in the regulation: *unacceptable*, *high*, *limited*, and *minimal* risk. Following the proportionate risk-based approach, riskier AI systems and applications are subject to stricter rules.

For example, AI applications in the category of ‘unacceptable risk’ are prohibited. These include applications that use biometric data to infer sensitive characteristics, engaging in non-targeted scraping of facial images from the internet to create facial recognition databases, or the use of emotion recognition in the workplace and in education (Article 5). Moreover, developers of high-risk AI systems must show that their models are safe, transparent, non-discriminatory, explainable to users, and adhere to privacy regulations (Article 6).

While the risk-based approach adopted in the AI Act has received positive response in the scholarly literature and wider governance community, it has not been immune to criticism. For example, it has been pointed out that the approach is too static, as it does not consider how hazard sources, vulnerability profiles, and fundamental values can and often do interact (AI4Belgium, 2021; Novelli et al., 2023; Kurian, 2024).

A more fundamental line of criticism of the Act, based on an appreciation of the different kinds of impacts that new technologies can have (hard and soft impacts; see Box 2), underlines the relevance of pursuing a social disruptiveness-based approach. Indeed, the traditional focus on quantifiable and direct harms in technology assessment overlooks the transformative soft impacts of new technologies, such as changes in values, norms, and social practices. These soft impacts, which are often qualitative and causally opaque, can have deep implications for humans and society, making it necessary to complement regulatory oversight with approaches that address these broader societal transformations. A comprehensive AI governance framework should therefore consider both hard and soft impacts, emphasising the need for a social disruptiveness-based approach to effectively guide the normative implications of AI technologies.

### Hard and Soft Impacts of Technology

The distinction between hard and soft impacts of technologies stems from critiques of Technology Assessment (Grunwald, 2009), which has been argued to rely on too narrow an assessment of impacts that (1) tend to be quantifiable, (2) tend to lead to clear harm, and (3) are directly caused by the emergence of a new technology. Impacts often concern concrete and observable consequences of the application of a specific technology, which are clearly recognisable and quantifiable, as they may involve physical changes or effects that can be competently assessed, e.g., pollution, health risks, and physical damages (Swierstra & te Molder, 2012). These so-called ‘hard impacts’ of technology (Swierstra, 2015) tend to receive the most attention in technology assessment. Yet, causal connections between technology and impact are frequently complex and indirect; technologies often have impacts that are not readily quantifiable; and there may be substantial uncertainty or ambiguity about harms. These consequences of new technologies have been characterised as ‘soft impacts’ (Swierstra, 2015), emphasising their less concrete nature.

Soft impacts include the ways in which technology can prompt individual or collective values, norms, social practices, and concepts to change (van der Burg, 2009; Swierstra & te Molder, 2012; Nickel et al., 2022; van de Poel, 2022a; Poel, 2022b). While soft impacts are easily overlooked in assessments with a narrow focus on risk, they can have deep and transformative implications for human life and society (Horowitz, 2020; König & Wenzelburger, 2020; Friedman, 2023). Consider the development of smart and connected information and communication devices—in particular smartphones and laptops—which have had a major influence on human behaviour and social organisation around the globe. Plausibly, this development has contributed to the changing nature political communication; it has served to further integrate the global economy, overcoming obstacles of distance; it has foregrounded new values, such as digital well-being, and facilitated shifting norms, for instance about online availability (Burr et al., 2020; Vanden Abeele, 2021; Dennis & Ziliotti, 2023). Soft impacts are at the core of these developments; indeed, they are central to the ‘deep impacts’ of socially disruptive technologies. Their importance is hard to overestimate, and it seems plausible that the widespread adoption of AI will similarly bring transformative soft impacts in its wake.

Should the more qualitative, causally opaque and normatively ambiguous implications of AI technologies be subject to regulatory oversight? We do not think this is always feasible. They are accompanied by substantial epistemic and moral uncertainty, which precludes straightforward regulatory measures. Yet instruments of governance are broader than regulation alone. Since soft impacts may have far-reaching implications that can be transformative to society, complementary approaches to oversight and anticipation are clearly needed. Such governance may involve setting up new institutions, stimulating or disinhibiting a certain set of norms, values, and ethical principles, and fostering societal debate in ways that go beyond regulation.^2^

In sum, given that hard and soft impacts of new technologies are both of great significance to society, a comprehensive AI governance framework should neglect neither. Accordingly, we will now argue that the AI Act’s risk-based approach should be complemented with a social disruptiveness-based approach, which places more emphasis on ‘softer’ impacts that nonetheless need to be considered for the normative guidance of AI’s socially disruptive implications.

## Social Disruptiveness-Based Approach

A social disruptiveness-based approach strives to identify the transformative implications that AI may have on humans, societies, and the environment in the near-, mid- and long-term future. Unlike a risk-based approach, which foregrounds the adverse impacts of AI, a social disruptiveness-based approach is not inherently focused on negative or positive implications. As a first approximation, ‘disruptiveness’ should be understood as describing the conjunction of technology’s soft and hard impacts, without any inherent positive or negative connotation.

The starting point of the social disruptiveness-based approach is that many of the implications of AI technologies are couched in uncertainty and ambiguity. Their social impacts may turn out for the better or for the worse, but they are rarely exclusively positive or negative. Some social disruptions brought about by AI are associated with straightforward risks of harm and call for regulation. Other disruptions do not call for regulation, but rather for normative re-orientation. For instance, generative AI appears to challenge the notion of ‘authorship’ and associated rights and recognition, and it is a matter of normative debate how this challenge should be resolved (van Woudenberg et al., 2024). Still other disruptions warrant the erection of new non-legal institutions that can help to guide the emergence of AI in a responsible manner. Although rare, some social disruptions will be so extreme that they should be avoided.

### Benefits of a Social Disruptiveness-Based Approach

Approaching AI governance through the lenses of the social disruptiveness of AI enables one to identify and highlight features and dimensions of impact beyond those primarily targeted by the AI Act and legal regulation at large. Indeed, limiting AI governance to a risk-based approach may lead to the soft impacts of AI being overlooked, such as indirect disruptions to society, norms, and values (see Sect. “Hard and Soft Impacts of Technology”).

On the one hand, this is to be expected. Indeed, it would be both uncharitable and unreasonable to expect the AI Act to comprehensively and satisfactorily account for such forms of impact, which often lie beyond the scope of legal regulation. On the other hand, such impacts are mentioned in the White Paper on AI as being worthy of consideration (European Commission, 2020). Complementing the AI Act risk-based approach with a social disruptiveness framework allows one to highlight impacts of AI that are not easily captured by legal regulation, but that are nonetheless socially relevant in that they raise serious concerns that require an ethical response.

Moreover, soft impacts also become relevant from a broader perspective. Indeed, the AI Act explicitly accounts for the need for an (EU) value-centric approach, which is theoretically dynamic and compatible with changes in such values. Importantly, this is not a unique feature of the AI Act, but can be generalised to other EU legal measures. Therefore, a social disruptiveness-based perspective may allow legal practitioners to be more mindful of the impact of AI with respect to changes in societal values. Overall, adopting a social disruptiveness-based approach to the governance of AI systems and applications allows us to account for impacts that are less tangible but that stand to be just as (if not more) socially transformative.

### Dimensions of Social Disruptiveness

Social disruption is not an all or nothing affair. The disruptiveness of a technology is relative to a given context and may change over time. Nonetheless, there are certain general indicators by virtue of which a technology may be deemed more or less disruptive. Following Hopster (2021), we propose that the social disruptiveness of AI can be estimated across different dimensions, namely the depth (ranging from shallow to deep disruptions, loosely comparable to the ‘soft impacts’), breadth, and valence of the disruption, its ethical significance, the uncertainty it generates (both epistemic and normative; see Mittelstadt et al., 2015), the pace of change, the irreversibility of the disruption, and the differential impact (see Table 2). Importantly, we do not claim that the proposed dimensions exhaustively capture all possible dimensions of social disruptiveness. Rather, we intend to offer a structured framework that captures what we take to be the most salient dimensions of impact at present which, in our view, best facilitate a nuanced and operationalisable analysis of the social disruptiveness of AI systems.

We also recognise that there will be some overlap among these dimensions. For instance, a high differential impact may also carry significant ethical weight, a rapid pace of change may exacerbate regulatory uncertainty (cf. discussions of the ‘pacing problem’ in law, and deep disruptions may also yield moral uncertainty, insofar as they cast doubt on moral norms and values. These overlaps are not problematic for our purposes, as the aim is not to delineate mutually exclusive dimensions, but to offer a heuristic structure for analysis. In addition to these dimensions, we wish to highlight the role of societal preparedness as a mediating variable that influences how different dimensions of social disruptiveness manifest. For example, while rapid change can create urgent governance challenges, the degree to which a society is prepared for such change—e.g., through existing institutions, regulatory frameworks, and public awareness—can significantly impact the extent of disruptiveness experienced. Ultimately, the dimensions we distinguish are not intended to serve as a rigid taxonomy that precludes further interpretation and refinement, but as a defeasible starting point for thinking about the socially disruptive implications of emerging technologies.

**Table 2:** Social disruptiveness-based approach: dimensions of impact

Dimension of impact	Description
*Depth of disruption*	The extent to which the SDT affects deeply-held beliefs, concepts, values, norms, institutions, and basic human capacities
*Breadth of disruption*	The range and variety of societal domains affected by an SDT
*Valence*	The emotional or affective impact generated by the SDT, encompassing both positive and negative dimensions
*Ethical significance*	The extent to which the SDT leads to first-order moral concerns, on moral norms, values, and principles
*Uncertainty*	The extent to which the implications of the SDT are cast in epistemic uncertainty (i.e., related to the lack of definitive or complete knowledge) and normative uncertainty (i.e., related to unsettled or contested normative frameworks for evaluation and action)
*Pace of change*	The rapidity of the change provoked by the SDT, and the associated degree of societal preparation and urgency of a societal response
*Irreversibility*	The extent to which the implications of the SDT are likely to be long-lasting and lead to lock-in
*Differential impact*	The extent to which the SDT differently affects different groups within and across societies

### Tentative Operationalisation

The social disruptiveness-based approach can be operationalised by applying the indicators of disruptiveness described in Table 2 to a given technology or application. Our present aim is not to provide a fully-fledged application of the approach to AI-systems, which would require rigorous empirical methodologies, incorporating both qualitative and quantitative analyses to evaluate the dimensions of impact in a systematic and context-sensitive manner. Instead, the following discussion and examples serve to clarify the theoretical presuppositions of the approach; the figures presented are speculative and used for illustrative purposes.

**Fig. 1:**
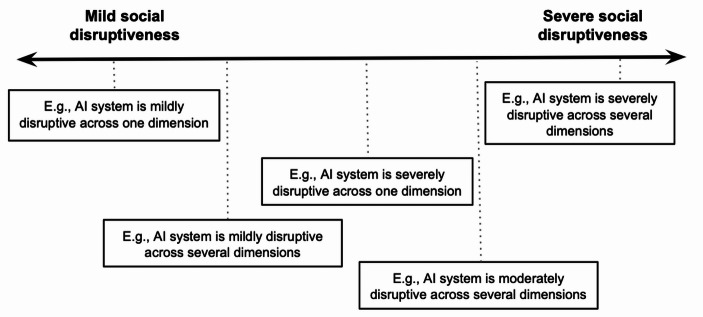
Social disruptiveness of AI systems (tentative illustration of the phenomenon)

Importantly, social disruptiveness is not a binary property but comes in degrees. The presence or co-occurrence of certain features makes it possible to classify a system on a scale extending from minimally socially disruptive to maximally socially disruptive (e.g., minimally SD, mildly SD, moderately SD, severely SD, maximally SD). Importantly, this means that, while AI systems or applications giving rise to severe social disruption alongside one or more dimensions will be considered severely socially disruptive, AI systems or applications may also be deemed severely socially disruptive if they lead to milder forms of disruption alongside several dimensions (see Fig. 1, illustrating a cross-section of the continuum of social disruptiveness).

As already discussed, a social disruptiveness-based approach can provide a more fine-grained account of what makes an AI system or application (potentially) harmful, and on which grounds, compared to a risk-based approach, as the social disruptiveness of a technology is not measured in binary terms, but rather in degrees, based on a combination of the presence and intensity of factors deemed to be indicative of social disruptiveness. Furthermore, this approach also allows potentially positive impacts to be captured. Therefore, a social disruptiveness approach is more finely calibrated compared to the risk-based approach, and can spot potential issues that the latter may overlook. For example, the AI Act risk-based approach focusses on few problematic features manifesting to a substantial degree. Conversely, as illustrated in Fig. 1, an AI system that displays several “socially disruptive features” to a low extent may be identified as moderately disruptive by a social disruptiveness-based approach.

**Fig. 2:**
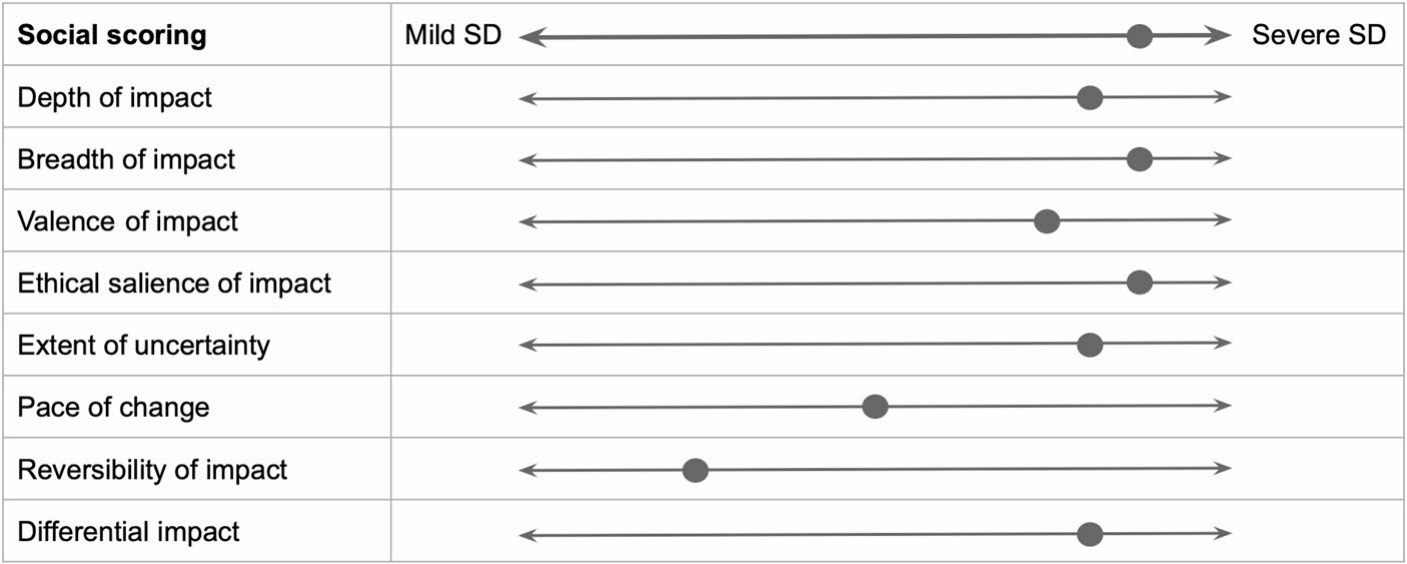
AI systems intended to be used for social scoring: illustration of social disruptiveness

**Fig. 3:**
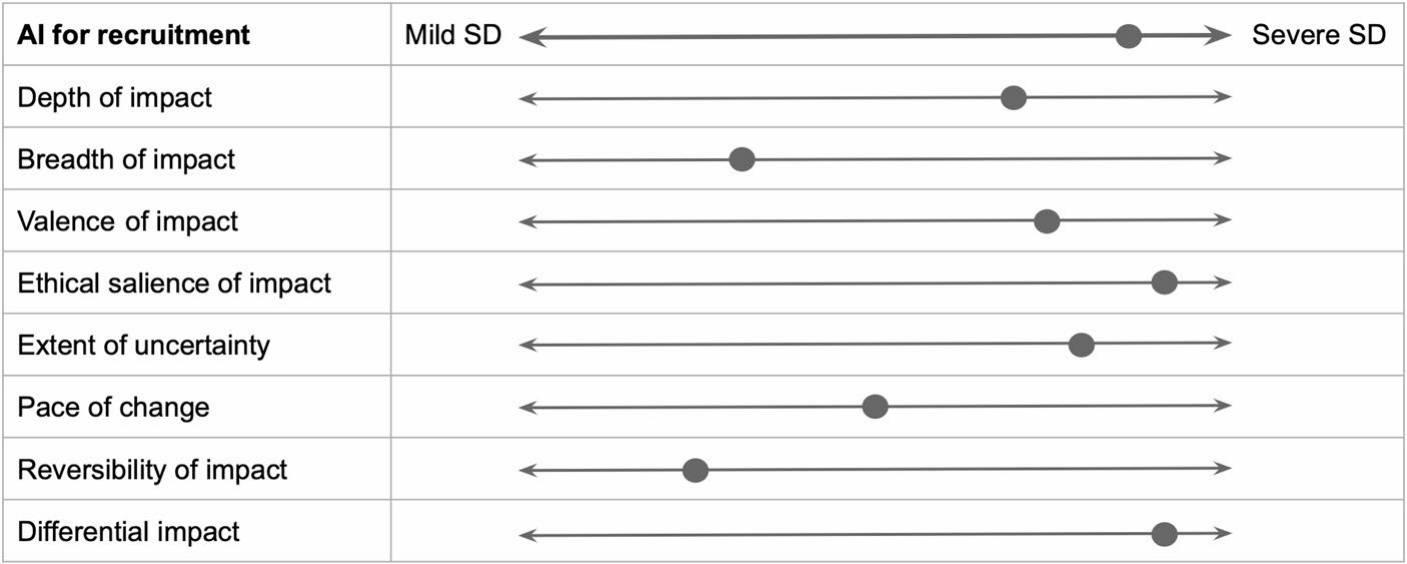
AI systems intended to be used for recruitment purposes: illustration of social disruptiveness

**Fig. 4:**
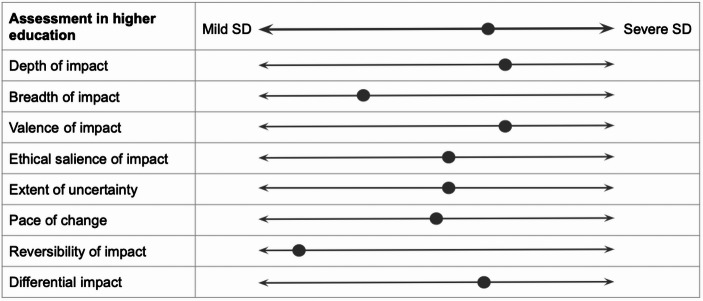
AI systems intended to be used for assessment in higher education: illustration of social disruptiveness

Consider the examples illustrated in Figs. 2 and 3, and 4, illustrating a speculative social disruptiveness assessment of AI applications used for social scoring (categorised as unacceptable risk following Article 5 of the Act), for recruitment purposes (belonging to high-risk AI systems following Article 6 of the Act), and for assessment in higher education (for which no compulsory norms are prescribed in the Act), alongside eight dimensions of impact.

Similarly to the risk-based approach, the social disruptiveness-based assessment of the first two applications identified them as (undesirably) severely socially disruptive. However, in the case of social scoring, AI scores high on seven dimensions, medium on one dimension, and low on one dimension. Conversely, AI for recruitment scores high on five dimensions of impact, medium on one dimension, and low on two dimensions. This allows us to gain a more fine-grained understanding of why it is reasonable to regulate social scoring more strongly than AI for recruitment. Moreover, the social disruptiveness-based assessment of AI for assessment in higher education highlights several dimensions of impact alongside which this application scores moderately. This suggests that, despite scoring lower on the social disruptiveness scale compared to AI systems belonging to the unacceptable or high-risk category, AI applications for which no mandatory norms are laid down in the AI Act may still deserve further societal attention.

### Risk-Based Vs. Social Disruptiveness-Based Approach

How does the social disruptiveness-based approach differ from the risk-based approach of the AI Act? One important difference is that the presence of social disruption is neither inherently problematic, nor necessarily indicative of (actual or expected) societal harms. The social disruptiveness approach identifies positive and negative social disruptions brought about by AI systems. Yet a high degree of disruptiveness may also be indicative of a need for societal debate on permissibility and precaution, on the responsibilities of disruptive innovators, as well as reflection on the appropriateness of existing institutions and human practices that can guide new technologies. Accordingly, an assessment of social disruptiveness can serve to prioritise applications for which risks are uncertain and ambiguous, yet for which the need for societal discussion and ethical reflection is evident. As such, assessing social disruptiveness serves as a tool for prioritising concerns about technology in anticipatory governance.

It should also be noted that, while high-risk AI systems and applications and severe social disruptiveness may often co-occur or overlap in some salient respect, such that it is possible (and perhaps reasonable to expect) that an AI system deemed highly risky from a risk-based approach will also score highly on the social disruptiveness scale, it does not follow from the fact that an AI system is highly risky that is is also severely socially disruptive (or vice versa). This adds an important nuance to the discussion surrounding the impacts of AI. Ultimately, the risk-based approach and the social disruptiveness-based approach should not be considered mutually incompatible but should be understood as complementary.

## Conclusion

In this paper, we considered the AI Act in a broader framework for the governance of AI systems in the European Union. We illustrated how, while the AI Act’s risk-based approach provides a robust legal foundation for the regulation of AI systems, it is insufficient on its own to address the multifaceted impacts of AI systems on society at large. We illustrated how, beyond the legal sphere, the risk-based approach implemented in the AI Act can be supplemented with other governance measures accounting for the impact of AI on broader socio-technical landscapes. Specifically, we proposed that a closer connection should be established between the legal regulation and the ethical governance of AI, specifically by complementing the AI Act risk-based approach with a social disruptiveness-based approach that focuses on the potential for AI to be socially disruptive. We argued that a social disruptiveness-based approach can offer a more nuanced understanding of AI’s potential to impact and transform society at large, while considering both positive and negative impacts of AI systems and their ethical ramifications. Ultimately, integrating such a perspective by supplementing the AI Act risk-based approach with a social disruptiveness-based approach allows us to shed light on AI applications, as well as other socially disruptive technologies, that warrant societal debate and ethical reflection, thus ensuring a more comprehensive and dynamic ecosystem of governance.

## Data Availability

Not applicable.

## Footnotes

1 https://esdit.nl/.

2 The point could be made that, while the regulation of soft impacts is often challenging due to their qualitative and normatively ambiguous nature, there may be cases where regulatory intervention is feasible—particularly when soft impacts manifest as indirect but discernible harms (e.g., algorithmic discrimination in hiring practices) or when they have significant implications for fundamental rights and democratic stability. However, in many cases, governance of soft impacts may be better served by non-regulatory mechanisms, such as ethical oversight bodies, public deliberation, and institutional adaptation.
